# Ultrasound-Guided Rectus Sheath Block With Monitored Anesthesia Care for Necrotic Umbilical Hernia Repair in a Patient With Severe Liver Failure and Refractory Ascites: A Case Report

**DOI:** 10.7759/cureus.80433

**Published:** 2025-03-11

**Authors:** Keisuke Nakazawa, Risyun Ishikawa, Takahiro Suzuki

**Affiliations:** 1 Anesthesiology, Nihon University School of Medicine, Tokyo, JPN

**Keywords:** antibiotic-resistant infection, ascites, coagulopathy, dexmedetomidine, hepatic encephalopathy, liver failure, monitored anesthesia care, rectus sheath block, regional anesthesia, umbilical hernia

## Abstract

Patients with severe liver dysfunction present significant perioperative challenges, including the risk of postoperative cognitive dysfunction (POCD) and hepatic encephalopathy (HE), after general anesthesia. While avoiding general anesthesia and deep sedation is crucial for early recovery in this patient population, neuraxial block techniques are often contraindicated due to coagulation disorders.

A 73-year-old male patient (190 cm tall, weighing 77 kg) with Child-Pugh C cirrhosis (score 10), coagulopathy (platelets 90,000/μL, prothrombin time (PT) activity 47%), and complex medical history, including treated hepatocellular carcinoma, renal cancer, and bladder cancer, underwent necrotic umbilical hernia repair. The patient, classified as American Society of Anesthesiologists (ASA) physical status IV with a Model for End-Stage Liver Disease (MELD) score of 19, had been hospitalized for two months due to an umbilical hernia infection refractory to antibiotic therapy. After careful preoperative assessment, we selected monitored anesthesia care (MAC) as the preferred anesthetic approach due to the patient's high surgical risk. We performed a bilateral rectus sheath block (RSB) using diluted ropivacaine (0.15%, total 80 mL) with epinephrine (15 μg). Sedation was achieved using dexmedetomidine without a loading dose, supplemented with midazolam and low-dose remifentanil. This approach allowed us to maintain spontaneous breathing while providing adequate analgesia and patient comfort. The surgery was completed successfully with stable hemodynamics and respiratory functions. Throughout the procedure, hemodynamic parameters remained within 20% of baseline values, and bispectral index (BIS) values were maintained between 65 and 80, indicating appropriate sedation depth without excessive anesthetic administration.

Ultrasound-guided RSB combined with carefully titrated MAC provides safe and effective anesthesia for umbilical hernia repair in patients with severe liver dysfunction. This approach maintains spontaneous breathing, delivers effective analgesia for somatic and visceral pain, and facilitates clearer differentiation between residual anesthetic effects and worsening HE postoperatively. When coagulopathy precludes neuraxial techniques, this pharmacokinetically informed strategy offers a valuable alternative for high-risk abdominal wall procedures.

## Introduction

Anesthetic management of patients with severe liver dysfunction presents unique challenges. Perioperative deterioration of liver function can lead to postoperative cognitive dysfunction (POCD) and hepatic encephalopathy (HE) after general anesthesia, making it difficult to differentiate between residual anesthetic effects and worsening encephalopathy [1,2]. Minimizing drug administration while providing adequate analgesia and sedation is crucial in these patients for early recovery protocol. The optimal anesthetic approach for abdominal wall procedures in patients with end-stage liver disease that facilitates early ambulation and recovery remains unclear and is a subject of ongoing debate.

Umbilical hernias are common complications in cirrhotic patients with ascites [3], occurring in approximately 20% of this population [4]. These hernias develop due to increased intra-abdominal pressure from ascites, which leads to protrusion through the umbilical fascial defect. Complications such as strangulation, incarceration, and hernia skin breakdown with leaking ascites are well-documented risks in these patients, with mortality rates significantly higher than in the general population [5].

When these hernias become infected, conservative medical management with antibiotics is typically attempted first. However, in cases where infection is refractory to antibiotic therapy, surgical intervention becomes necessary despite the high perioperative risks, as untreated infection can lead to sepsis, peritonitis, and further hepatic decompensation [6].

Monitored anesthesia care (MAC) requires careful consideration of patient safety and appropriate patient selection [7]. For patients with hepatic dysfunction, MAC offers advantages over general anesthesia by facilitating clearer postoperative neurological assessment, helping clinicians differentiate between residual anesthetic effects and worsening HE. This approach may reduce the risk of POCD. A recent meta-analysis demonstrated that dexmedetomidine significantly reduced POCD incidence compared with placebo (12.9% vs. 27.7%) and sevoflurane (24.0%) in noncardiac surgery [8].

Additionally, neuraxial techniques such as spinal anesthesia or epidural block are often contraindicated in these patients due to coagulopathy [9]. In this context, ultrasound-guided rectus sheath block (RSB) has emerged as a valuable technique that can be combined with MAC to provide excellent analgesia for abdominal wall procedures.

Providing adequate anesthesia for trunk procedures with conscious sedation remains challenging for anesthesiologists. The combination of ultrasound-guided peripheral nerve blocks with alpha-2 agonists like dexmedetomidine and diluted low-dose remifentanil can maintain spontaneous breathing while providing optimal surgical conditions comparable to general anesthesia, with high patient satisfaction. Recent multicenter trials have shown that dexmedetomidine is an effective baseline sedative, providing greater patient satisfaction, reducing opioid requirements, and causing less respiratory depression compared with placebo [10].

This report describes a case of successful necrotic umbilical hernia repair using RSB with MAC in a patient with Child-Pugh C cirrhosis, emphasizing how this approach can preserve hepatic function while ensuring optimal surgical conditions.

## Case presentation

A 73-year-old male patient (190 cm tall and weighing 77 kg) with Child-Pugh C cirrhosis (score 10, HE grade I, massive ascites, total bilirubin 1.4 mg/dL, albumin 2.4 g/dL, prothrombin time (PT) activity 47%) presented with a 5-cm necrotic umbilical hernia. Preoperative laboratory findings are summarized in Table 1.　

**Table 1:** Preoperative laboratory results of a 73-year-old male patient with Child-Pugh C cirrhosis WBC: white blood cell, RBC: red blood cell, CRP: C-reactive protein, AST: aspartate aminotransferase, ALT: alanine aminotransferase, PT: prothrombin time, INR: international normalized ratio, eGFR: estimated glomerular filtration rate.

Parameter	Patient's value	Reference range
WBC	5,800/μL	3,500-9,000/μL
RBC	3.6 × 10^6^ /μL	4.0-5.5 × 10^6^/μL
Hematocrit	36%	40%-54%
Platelets	90 × 10^3^/μL	150-450 × 10^3^/μL
Total protein	5.6 g/dL	6.0-8.0 g/dL
Albumin	2.4 g/dL	3.5-5.0 g/dL
CRP	24.8 mg/dL	<0.3 mg/dL
Sodium	137 mmol/L	135-145 mmol/L
Potassium	4.8 mmol/L	3.5-5.0 mmol/L
Chloride	104 mmol/L	98-107 mmol/L
Creatinine	1.9 mg/dL	0.6-1.2 mg/dL
eGFR	28.2 mL/min/1.73 m^2^	>90 mL/min/1.73 m^2^
AST	26 U/L	10-40 U/L
ALT	14 U/L	5-45 U/L
Total bilirubin	1.4 mg/dL	0.2-1.0 mg/dL
PT activity	47%	70%-130%
PT-INR	1.6	0.9-1.1

The patient's medical history was significant for alcoholic liver cirrhosis (initially diagnosed in 2010), hepatocellular carcinoma (15-mm lesion in the S5 segment treated with radiofrequency ablation in 2022), left partial nephrectomy for renal cancer (2019), and muscle-invasive bladder cancer with ileal neobladder construction (2010). All malignancies were in remission, with no evidence of recurrence. The patient was under regular follow-up for esophageal varices, pleural effusion, and ascites management.

Prior to surgery, the patient had been hospitalized for two months to investigate a fever and treat infection and ascites. The treatment regimen included antibiotics (meropenem 1 g twice daily) for the infected umbilical hernia, diuretics, and cell-free and concentrated ascites reinfusion therapy (CART) at 10 L per session for ascites control.

The necrotic area expanded despite aggressive antibiotic therapy, with purulent discharge and elevated inflammatory markers (WBC 5,800/μL, CRP 24.8 mg/dL). After a multidisciplinary discussion, it was concluded that a complete resolution with continued medical management was unlikely, and the patient faced a significant risk of sepsis and peritonitis. Therefore, despite the high perioperative risks associated with his severe liver dysfunction, surgical intervention was deemed necessary.

Given the patient's complex medical history, including multiple prior abdominal surgeries, renal impairment (eGFR 28.2 mL/min/1.73 m²), and advanced liver disease with coagulopathy, he was classified as American Society of Anesthesiologists (ASA) physical status IV. The Model for End-Stage Liver Disease (MELD) score was 19, indicating a high risk of perioperative morbidity and mortality.

Given the patient's severe liver dysfunction and the need to minimize drug administration to differentiate between residual anesthetic effects and worsening HE postoperatively, we opted for MAC with RSB. This approach would maintain spontaneous breathing while providing adequate surgical conditions.

**Figure 1:**
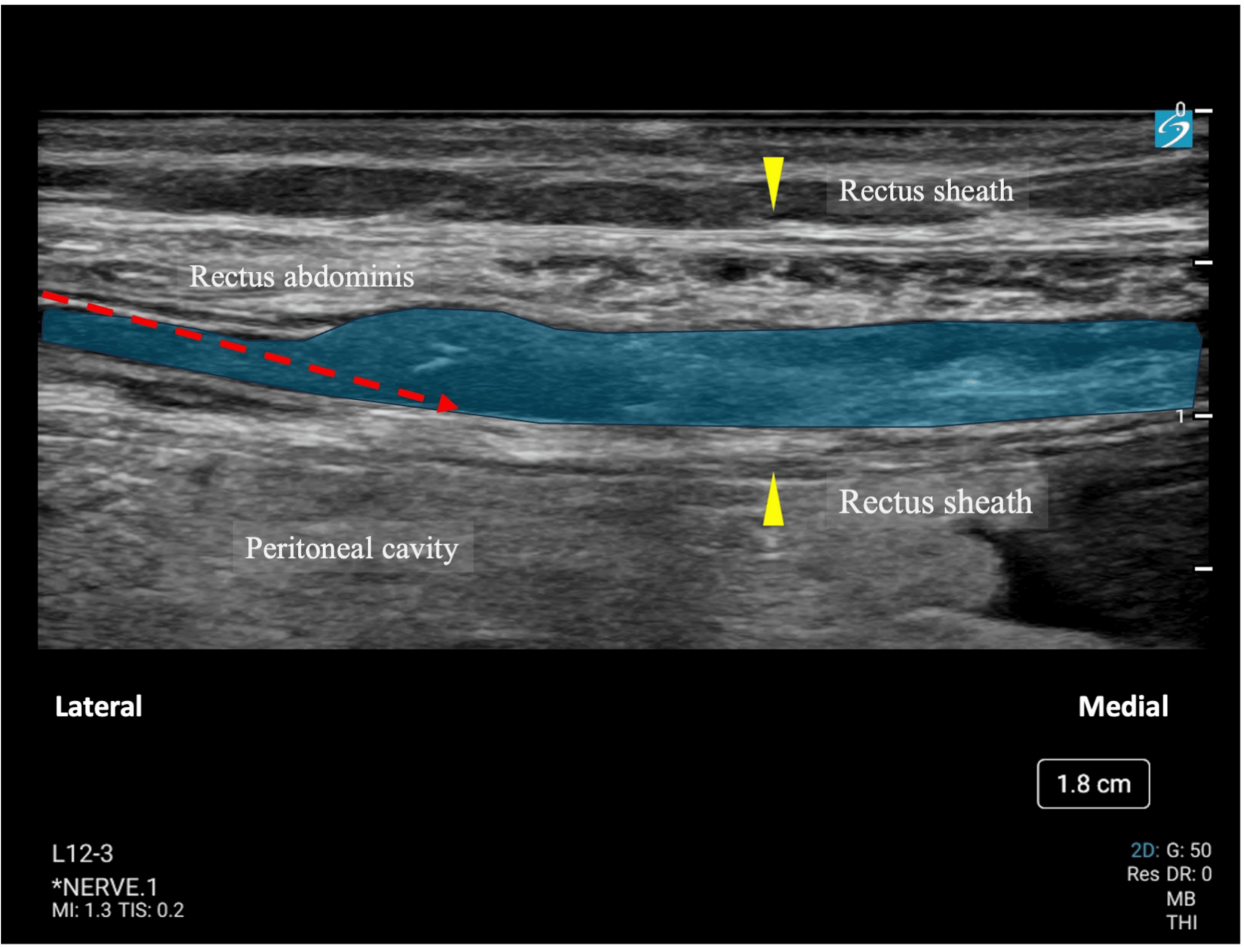
Ultrasound image during rectus sheath block The blue-shaded area represents the local anesthetic spread between the rectus abdominis muscle and posterior rectus sheath. The red dashed line demonstrates the needle trajectory. Yellow arrowheads point to the hyperechoic rectus sheath, with the upper arrowhead indicating the anterior rectus sheath and the lower arrowhead indicating the posterior rectus sheath. The peritoneal cavity is visible below the posterior rectus sheath.

With standard monitoring devices used in general anesthesia (electrocardiogram, noninvasive blood pressure, pulse oximetry, capnography via sampling line integrated into the oxygen mask, and bispectral index (BIS) monitoring), we initiated oxygen administration at 5 L/min via face mask. Under ultrasound guidance using a Sonosite SII ultrasound system (FUJIFILM Sonosite, Inc., Bothell, WA, USA) with a high-frequency linear transducer, we administered 0.15% ropivacaine (total 80 mL) with epinephrine (15 μg) in divided doses bilaterally between the rectus abdominis muscle and posterior rectus sheath. The procedure was performed using a Stimuplex® Ultra 360® 22 Ga. x 3-1/8 in. (80 mm) Insulated Echogenic Needle with a 30° Bevel and Extension Set (B. Braun Medical Inc., Bethlehem, PA, USA) with an in-plane approach (Figure 1).

**Figure 2:**
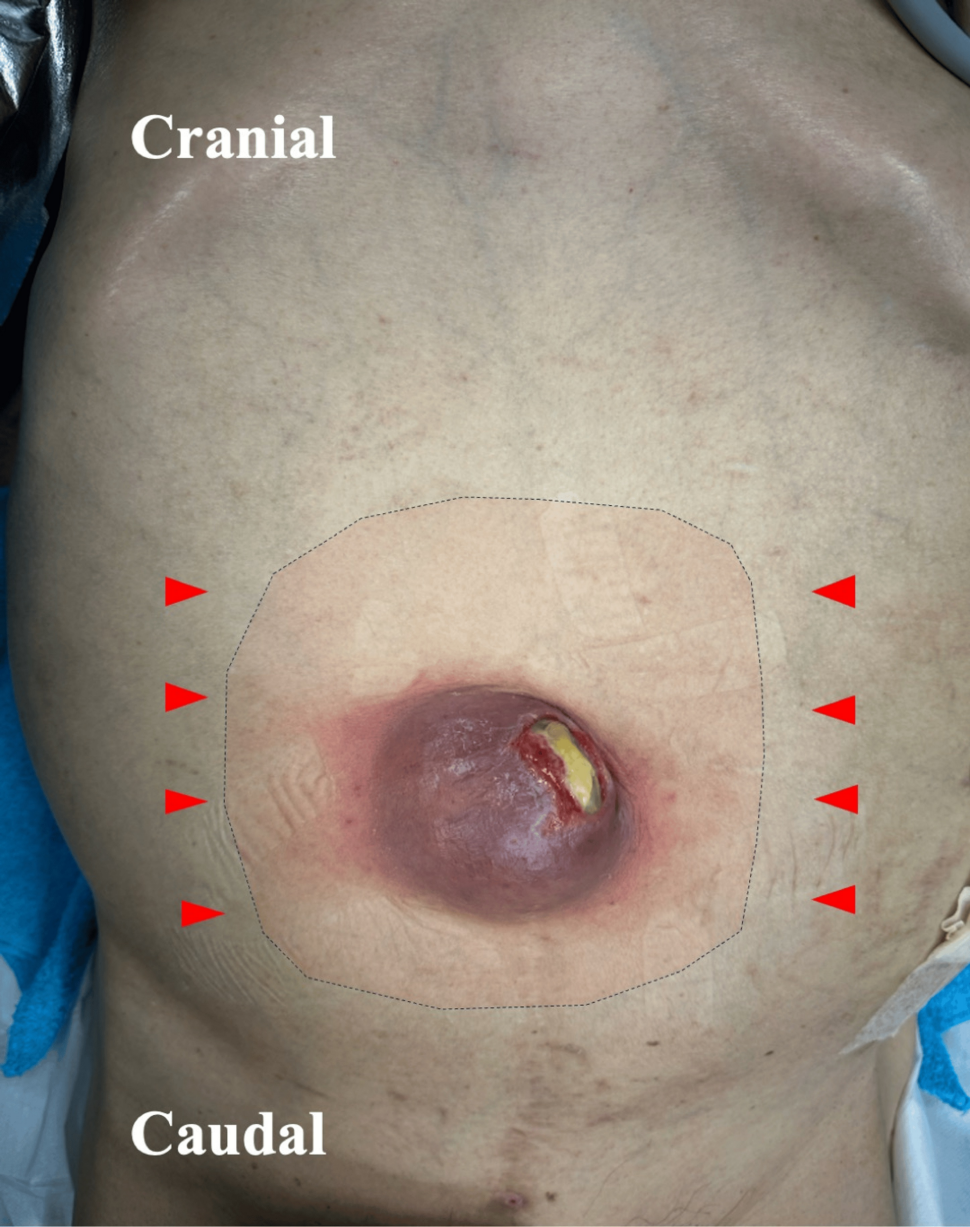
The infected necrotic umbilical hernia before surgery and the region of sensory blockade The photograph shows the central lesion with purulent discharge and the distended abdomen due to ascites. Red arrowheads: rectus sheath block (RSB) injection sites at the anterior axillary line. Pink-shaded area within the black dashed line: region of sensory blockade.

After confirming the sensory blockade using both alcohol swab cold testing and direct surgical field assessment with forceps, sedation was achieved using dexmedetomidine (0.2 μg/kg/h) without a loading dose, supplemented with midazolam (1 mg) and low-dose remifentanil (0.01 to 0.05 μg/kg/min) diluted to a concentration of 5 μg/mL and administered via a dedicated intravenous line (Figure 2).

The 87-minute surgery was completed successfully with stable hemodynamics under this MAC with RSB strategy. The surgical approach involved excision of the necrotic umbilical hernia without mesh placement. After making an elliptical incision around the umbilical erosion, the surgeons carefully dissected the hernia sac, which contained multiple layers of dark red fibrin membranes. The sac was meticulously separated from the surrounding tissues despite occasional rupturing of the hernia wall. The base of the hernia where it invaginated into the abdominal wall was ligated using 2-0 Tyeron sutures in three layers, with an additional transfixion suture to ensure secure closure. After confirming no ascitic fluid leakage with manual compression, the remaining portion of the hernia sac in the subcutaneous tissue was excised, followed by thorough hemostasis and irrigation with saline-soaked gauze. The procedure concluded with fascial closure.

**Figure 3:**
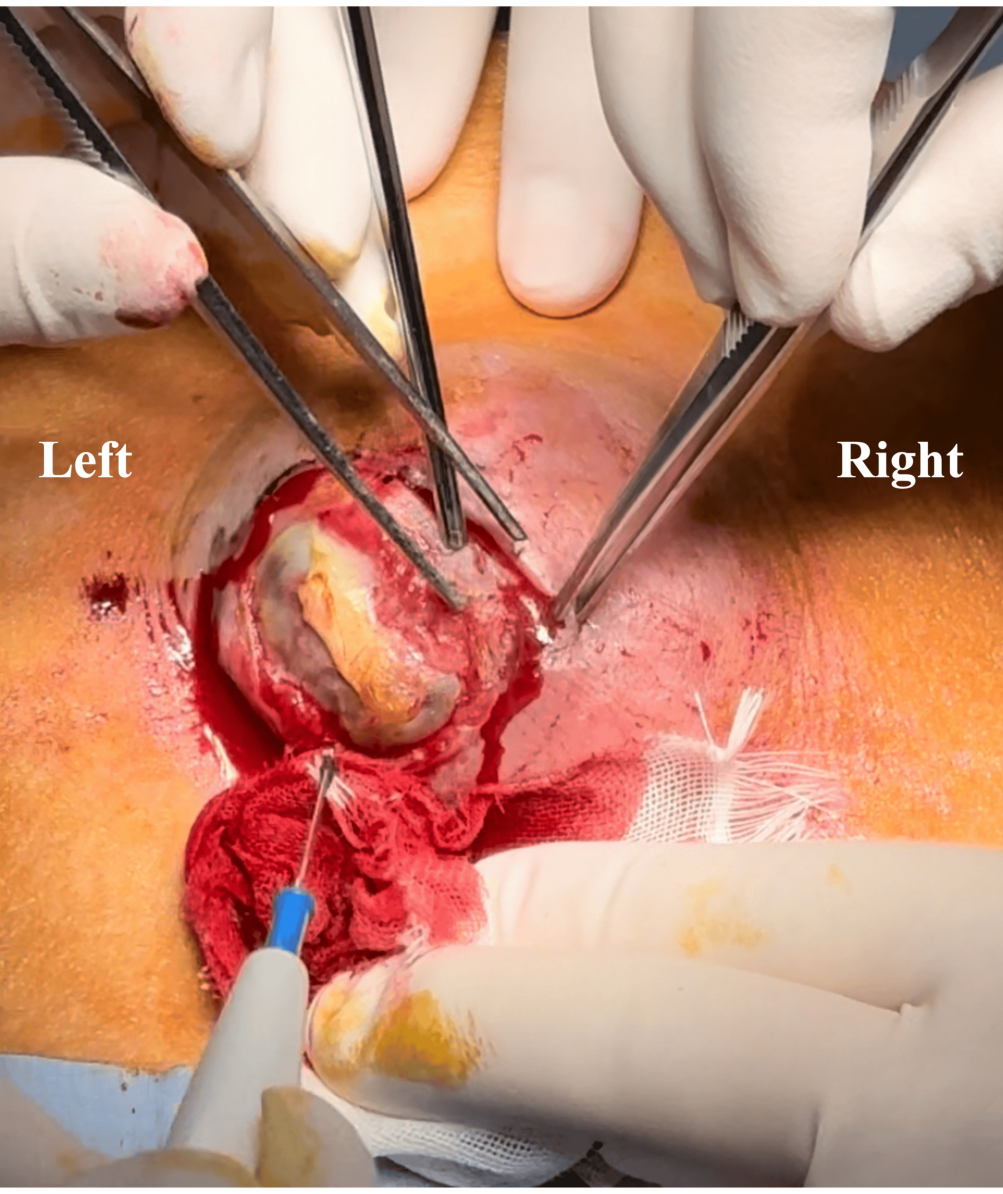
Intraoperative view of the necrotic umbilical hernia during surgical repair The image shows the hernia sac with surrounding tissue inflammation and debridement in progress. Surgical instruments are positioned to facilitate exposure of the defect. Despite the extensive tissue involvement, the patient remained comfortable throughout the procedure under RSB with MAC, demonstrating the effectiveness of this anesthetic approach even during the manipulation of inflamed peritoneal structures.

Throughout the procedure, we assessed the patient's respiratory status by verbal communication and capnography waveform analysis. The Ramsay Sedation Score remained stable between 2 and 4, and the respiratory rate was maintained at 8-15 breaths per minute. Notably, the patient did not experience any pain during peritoneal traction or ligation procedures (Figure 3).

Postoperatively, the patient recovered rapidly with excellent pain control and clear consciousness and resumed CART on postoperative day 3. The patient continued to improve steadily and was discharged home in stable condition on postoperative day 9.

## Discussion

Umbilical hernias are common complications in patients with liver cirrhosis, particularly in those with ascites, occurring in approximately 20% of such patients [3,4]. For patients with severe liver dysfunction, perioperative management focuses on maintaining liver function while providing adequate anesthesia. A key goal is to facilitate early ambulation and recovery, which can reduce complications associated with prolonged immobility and hospitalization in this high-risk population.

The management of infected umbilical hernias in cirrhotic patients presents a significant challenge with high morbidity and mortality. In a study by Cho et al., overall morbidity and mortality rates of 13.1% and 5.1%, respectively, were reported in patients with ascites and/or esophageal varices undergoing umbilical hernia repair, compared to 3.9% morbidity and 0.1% mortality in the general population [5]. In our case, although our patient had esophageal varices without definitive documentation of portal hypertension, the clinical presentation with severe liver dysfunction and ascites warranted similar considerations for perioperative risk assessment. While conservative treatment with antibiotics is typically the first-line approach, when the infection is refractory to medical therapy, as in our case, surgical intervention becomes necessary despite substantial perioperative risks. The decision to proceed with surgery requires careful balancing of risks of continued infection against risks of surgical and anesthetic complications in patients with severe liver dysfunction [1,2].

A postoperative key concern is the potential for worsening HE, which can be difficult to distinguish from residual anesthetic effects. Our approach prioritized minimizing drug administration while ensuring patient comfort and optimal surgical conditions.

Patients with Child-Pugh C cirrhosis present altered local anesthetic pharmacokinetics, necessitating careful dose adjustment [11]. In this case, we chose ultrasound-guided RSB over neuraxial techniques due to the patient's coagulopathy. Despite anatomical challenges from ascites, including thinning of the rectus abdominis muscle with the posterior sheath approximately 10 mm deep, ultrasonography enabled precise visualization of the epigastric artery, fascial planes, and muscle sheaths, ensuring safe and accurate needle placement.

For the block, we administered diluted ropivacaine (0.15%, total 80 mL) with epinephrine in divided doses bilaterally. This approach was supported by previous studies demonstrating that RSB using bupivacaine provides efficient analgesia with reduced postoperative complications in cirrhotic patients undergoing umbilical hernia repair [12]. While our approach differs slightly from the referenced study that used 0.25% bupivacaine (20 mL), we deliberately opted for a larger volume of more dilute ropivacaine (0.15%, 80 mL) for several reasons. First, our primary goal was to achieve complete surgical anesthesia under MAC with conscious sedation, not just postoperative analgesia, requiring comprehensive blockade across the entire surgical field. Second, the larger volume ensured adequate spread throughout the fascial plane, minimizing the risk of incomplete blockade. Third, considering the patient's severe liver dysfunction, we intentionally reduced the concentration to maintain the total ropivacaine dose at approximately half the maximum recommended amount, mitigating risks associated with altered drug metabolism. Finally, our clinical experience has shown that when supplemented with low-dose remifentanil, as used in this case, even dilute solutions (0.15% ropivacaine) can provide effective surgical anesthesia for these procedures.

We specifically selected ropivacaine for its reduced central nervous system and cardiac toxicity compared to bupivacaine [13]. Considering that ropivacaine clearance decreases by approximately 60% in patients with end-stage liver disease [11], resulting in elevated unbound drug fractions and prolonged elimination half-lives, we carefully calculated our dosage (1.55 mg/kg, approximately half the maximum recommended dose of 3 mg/kg) and utilized epinephrine to reduce systemic absorption. Given the patient's heightened risk for local anesthetic systemic toxicity, we prepared for immediate lipid resuscitation therapy as a precaution [14].

The effectiveness of our RSB approach was validated by Gurnaney et al., who demonstrated its superiority over local anesthetic infiltration for umbilical hernia repair [15], particularly relevant for cirrhotic patients where minimizing systemic analgesics is crucial. However, we recognized that while RSB effectively blocks abdominal wall pain transmitted through anterior branches of intercostal nerves, visceral pain components during peritoneal manipulation might remain. By supplementing the block with low-dose remifentanil, we achieved synergistic pain control, as evidenced by the absence of pain even during peritoneal traction and ligation, typically the most painful stimuli during hernia repair.

In our MAC protocol, intravenous dexmedetomidine and remifentanil were used to supplement the RSB. This anesthetic approach proved highly effective during peritoneal traction and ligation. While specific sedation protocols for umbilical hernia repair have not been extensively studied, the dexmedetomidine-remifentanil combination has been shown to reduce respiratory depression risk without increasing hemodynamic complications compared to propofol-remifentanil during MAC for various procedures [16].

In our patient, even during intense surgical stimuli, vital signs remained stable and no pain behaviors were observed. This confirms that RSB combined with low-dose remifentanil effectively managed both somatic and visceral pain without requiring general anesthesia. Dexmedetomidine's inherent analgesic properties helped reduce opioid requirements [17], while remifentanil offered a key advantage over conventional opioids like morphine or fentanyl: its metabolism by nonspecific blood and tissue esterases occurs independently of hepatic function, maintaining a consistent context-sensitive half-time regardless of liver impairment [18].

The success of this approach extended into the postoperative period, with our patient demonstrating favorable recovery. The patient was transferred to the ICU immediately after surgery (POD 0), resumed oral intake with a salt-restricted diet (6 g sodium) on POD 1, and was able to stand at the bedside and transfer from the ICU to the general ward on POD 2. Independent ambulation was achieved by POD 3. The subcutaneous Penrose drains were sequentially removed on PODs 2, 4, and 6, while the ascites drain was removed on POD 7. Throughout this period, the patient maintained clear consciousness and stable liver function. The patient was discharged home on POD 9, demonstrating the effectiveness of our anesthetic approach in facilitating relatively early recovery despite the high perioperative risk profile.

Recent studies have emphasized the importance of careful patient monitoring during MAC, since respiratory compromise from oversedation remains the most common complication [7]. To mitigate this risk, we employed a dual strategy: using a BIS monitor to assess consciousness level and prevent excessive sedation and selecting dexmedetomidine as our primary sedative due to its favorable respiratory profile [17]. Previous research has validated this approach, demonstrating that dexmedetomidine combined with midazolam under BIS monitoring provides safe and effective conscious sedation during procedures such as bronchoscopy [19]. Our remifentanil administration approach was designed to allow for flexible and rapid adjustments based on the patient's respiratory parameters. We diluted remifentanil to a concentration of 5 μg/mL and delivered it through a dedicated intravenous line. This dilution allowed for precise dosing control, with our dosing range of 0.01-0.05 μg/kg/min translating to manageable infusion rates (approximately 6 mL/h for a 50-kg patient at 0.01 μg/kg/min). This approach enabled immediate adjustments in response to real-time monitoring of respiratory rate, maintaining it between 8 and 15 breaths per minute as noted previously. Studies have confirmed this dosing range's tolerability even in high-risk patients with renal failure [20].

This case highlights how integrating regional anesthesia techniques with pharmacokinetically informed medication selection can transform the management of high-risk patients with severe liver dysfunction. By combining ultrasound-guided RSB with carefully selected sedatives, we achieved hemodynamic stability, effective pain control even during peritoneal manipulation, and rapid postoperative recovery with preserved liver function. This approach successfully addressed the dual challenge of providing adequate surgical conditions while facilitating clear differentiation between residual anesthetic effects and HE in the postoperative period.

## Conclusions

Ultrasound-guided RSB combined with carefully titrated MAC provides safe and effective anesthesia for umbilical hernia repair in high-risk patients with severe liver dysfunction and multiple comorbidities. This approach maintains spontaneous breathing, delivers effective analgesia for somatic and visceral pain, and facilitates clearer differentiation between residual anesthetic effects and worsening HE postoperatively. For ASA IV patients with high MELD scores where coagulopathy precludes neuraxial techniques, this pharmacokinetically informed strategy offers a valuable alternative for complex abdominal wall procedures even in the setting of antibiotic-resistant infection.
